# Rare variants in *TTC7A*,* ROCK2* and *LIMK2* suggest a role for the ROCK-signaling pathway in isolated intestinal malrotation

**DOI:** 10.1007/s00383-026-06442-2

**Published:** 2026-05-25

**Authors:** Karin Salehi Karlslätt, Maria Pettersson, Kristina Lagerstedt-Robinson, Ulla Ullberg, Anna Lindstrand, Agneta Nordenskjöld

**Affiliations:** 1 Department of Women’s and Children’s Health, Center for Molecular Medicine, Karolinska Institutet, Stockholm, Sweden; 2 Department of Pediatric Surgery, Karolinska University Hospital, BioClinicum J10:20, Solnavägen 30, Solna, 171 76 Stockholm, Sweden; 3 Department of Molecular Medicine and Surgery, Karolinska Institutet, Stockholm, Sweden; 4 Department of Clinical Genetics, Karolinska University Hospital, Stockholm, Sweden; 5 Department of Pediatric Radiology, Karolinska University Hospital, Stockholm, Sweden

**Keywords:** Intestinal malrotation, Intestinal rotation abnormalities, Midgut volvulus, Genome sequencing, *TTC7A*, *ROCK2*, *LIMK2*

## Abstract

**Purpose:**

This study aimed to identify genetic variants contributing to the development of early-diagnosed isolated intestinal malrotation.

**Methods:**

We conducted Genome sequencing on ten young children diagnosed with midgut volvulus due to intestinal malrotation who presented no other malformations or comorbidities. Our analysis focused on a panel of 442 genes previously associated with intestinal development, malrotation, ciliopathies and/or situs abnormalities.

**Results:**

In one male patent we discovered two heterozygous variants in *TTC7A*, c.433G > A p.(Ala145Thr) and c.1057G > A p.(Glu353Lys). Carrier testing revealed that he inherited both variants from his mother with a mild intestinal rotation abnormality. Additionally, we identified inherited variants in two other male participants, c.1802 A > T p.(Asp601Val) in *ROCK2* and, c.884 G > A p.(Arg295His) in *LIMK2*. These genes are part of a shared signaling pathway previously shown to cause intestinal malrotation in Xenopus when inhibited.

**Conclusion:**

Our findings suggest the potential involvement of *TTC7A*, *ROCK2* and *LIMK2-genes* in the pathogenesis of intestinal malrotation; through the ROCK-signaling pathway.

## Introduction

Intestinal Malrotation (IM) arises from incomplete rotation and fixation of the intestines during weeks 4–12 of gestation [1]. IM predisposes to midgut volvulus, a potential emergency and a life-threatening situation, or can cause chronic symptoms like abdominal pain, vomiting and faltering growth. The standard treatment is the Ladd´s procedure, in which the intestines are derotated if there is a volvulus, and then arranged in a non-rotational state to reduce the risk of volvulus recurrence. Traditionally IM manifests neonatally and with symptoms such as abdominal distension and biliary vomiting. However, it is increasingly recognized that IM can present at any age with some individuals remaining asymptomatic throughout their life. Conversely, prenatal volvulus has also been reported potentially leading to intestinal atresia if the fetus survives.

The exact incidence of IM is unknown but is estimated to range from 0.01 to 0.2%, with most cases being sporadic and isolated [2, 3]. There is a known male predominance in children with intestinal malrotation. Familial occurrences have been reported, mainly siblings [4–11], but also a few cases of larger families indicating an underlying molecular basis for IM [12–18] (Table 1).

**Table 1:** Published cases on familial IM

Reference	Description of family
Spitz, 1932 [15]	Cousins
Hadley, 1940 [16]	Parent–child
Rothenberg, 1958 [17]	Parent–child
Townes et al., 1962 [4]	Three sibs
Smith, 1972 [18]	Three generations
Carmi et al., 1981 [12]	Parent–child
Gibson, 1987 [5]	Two sibs, IM and jejunal atresia
Budd and Powley, 1988 [6]	Two brothers
Kern et al., 1990 [7]	Three brothers, IM, congenital short bowel and dysmotility
Stalker and Chitayat, 1992 [8]	Two sisters
Crowley and Bawle, 1996 [9]	Monozygotic twins
Erez et al., 2001 [13]	Three related families, where one of four, two of six and three of seven children had IM. Consanguinity, also short bowel presented
Beaudoin et al., 2005 [14]	Three sibs, mother with a minor duodenal anomaly
Thacker et al., 2009 [10]	Monozygotic twins, heterotaxy, one with IM
Nath & Corder, 2012 [11]	Two sibs

IM has also been linked to various chromosomal abnormalities and in combination with other malformations [3]. Previous studies have indicated an overrepresentation of pathogenic copy number variants (CNV) in IM patients with additional malformations or syndromic presentations [19]. Although associations with single genes have been reported in different syndromes as well as with other malformations [20–22], no causative single gene has been identified in isolated IM cases.

We conducted a pilot study with genome sequencing (GS) focusing on gene panels of ten children with an early and severe form of isolated midgut volvulus due to IM to investigate the presence of single gene disease-causing genetic variants.

## Materials and methods

### Editorial policies and ethical considerations

The study was approved by the Swedish Ethical Review Authority Dnr: 2013/944 − 31. The principles of the Declaration of Helsinki were followed and informed consent was obtained from all included patients and family members.

Patients were identified through ICD-codes Q433 or K562, from registers of children (0–15 years of age) treated at the Karolinska University Hospital (Stockholm, Sweden). The inclusion criteria were isolated IM predisposing to midgut volvulus with the duodenum positioned on the right side of the vertebral spine. Medical records were carefully reviewed, also radiologically, to conform the accuracy of the diagnosis (n = 138). Male:female ratio in this group was 2.1:1. Fifty % had other malformations and were excluded. Thirty-five had surgery before 1 year of age [23]. A cohort of ten patients, diagnosed from 1999 to 2012 with isolated IM and early onset midgut volvulus confirmed through surgery were included from those we could collect blood samples from. Blood samples were collected from relevant family members.

DNA was extracted from whole blood according to standard procedures.

Genomic sequencing (GS) was performed at Clinical Genomics, Stockholm, Sweden using the Illumina HiSeq X Ten, or NovaSeq 6000 platforms, using a 30 × PCR-free paired-end WGS protocol. Different categories of genetic variants were called using Mutation Identification Pipeline (MIP version 9.1.2) [https://github.com/Clinical-Genomics/MIP] [24]. The bioinformatic analysis includes calling of single nucleotide variants (SNVs), insertions and deletions (INDELs), short tandem repeats (STRs), uniparental disomies and structural variants (SVs) including deletions, duplications, inversions as well as insertions of mobile elements (MEI). To confirm findings, Sanger sequencing was used.

The GS-data was analyzed using Scout version 63.0, with reference genome GRCh37 (hg19), employing in silico gene-panels. Specifically, we analyzed an HPO-panel for IM (HP:0002566), heterotaxy (HP:0030853 (143 genes, Table 2a) and ciliopathies (204 genes, Table 2b). Additionally, we included an analysis of 103 other genes reported in the literature to be involved in intestinal development or previously linked to IM (Table 2c). We also evaluated the genes within CNVs reported in our recent study [19] and constructed a gene panel based on them (n = 13) (Table 2d). Finally, 22 genes were later selected for their interaction with or as paralogues to *TTC7A* and *TTC7B* (Table 2e). Altogether 442 unique genes were analyzed (summarized in tables 2a–e). Moreover*,* in four patients, who had not undergone Chromosomal microarray analysis (CMA) prior to GS, SVs in 1,989 genes involved in early development and ciliopathies, were assessed [19, 25]. GnomAD (https://gnomad.broadinstitute.org) and ClinVar (https://www.ncbi.nlm.nih.gov/clinvar/) were used to for annotation. Information on involved genes was collected from OMIM, GeneCards—The Human Gene Database (2021), and MalaCards (www.genecards.com) [26].

**Table 2:** Gene panels used in this study

(a) Gene panels from HPO (143 genes) HPO-terms: Intestinal malrotation HP:0002566, Heterotaxy HP:0030853
*ABL1, ACTA2, ACTG2, AMER1, ARID1B, ARVCF, ASXL1, BCOR, CCDC103, CCDC22, CCDC39, CCDC40, CCDC65, CCNO, CENPF, CFAP298, CFAP300, CFAP53, CFC1, CHRM3, CHST14, CLMP, COMT, CPLX1, CREBBP, CRELD1, CTBP1, DHCR24, DHCR7, DHODH, DNAAF1, DNAAF2, DNAAF3, DNAAF4, DNAAF5, DNAAF6, DNAH1, DNAH11, DNAH5, DNAH9, DNAI1, DNAI2, DNAJB13, DNAL1, DRC1, DSE, DYNC2H1, EP300, EYA1, FANCB, FARSB, FBN2, FGFR2, FGFRL1, FLI1, FLNA, FLNB, FOXF1, FOXJ1, GAS2L2, GAS8, GATA4, GATA6, GDF1, GP1BB, GPC3, GPC4, HDAC8, HIRA, HMGA2, HNRNPU, HYDIN, ISL1, JMJD1C, KAT6A, KDM3B, KDM6A, KLHL7, KMT2A, KMT2D, LBR, LEMD3, LETM1, LMOD1, LRP2, LRRC56, MCIDAS, MED12, MKS1, MMP21, MYH11, MYLK, MYRF, NEK1, NEK10, NELFA, NIPBL, NME8, NODAL, NOTCH2, NPHP3, NR2F2, NSD2, ODAD1, ODAD2, ODAD3, ODAD4, OFD1, PIGN, PKD1L1, PORCN, RAD21, RFX6, RPGR, RREB1, RSPH1, RSPH3, RSPH4A, RSPH9, SALL4, SEC24C, SETD5, SIX1, SLC12A2, SMC1A, SMC3, SMO, SPAG1, SPEF2, SPINT2, STK36, TBX1, TFAP2A, TP63, TTC12, TTC7A, UBE3B, UFD1, WASHC5, WNT4, ZFPM2, ZIC3, ZMYND10*
(b) Gene panel for ciliopathy (204 genes)
*ADGRV1, AGBL2, AHI1, AIPL1, ALMS1, ANKS6, ARL13B, ARL3, ARL6, ARMC4, ARMC9, ATXN10, B9D1, B9D2, BBIP1, BBS1, BBS10, BBS12, BBS2, BBS4, BBS5, BBS7, BBS9, BMP1, BRAF, C21orf59, C2CD3, C2orf71, C5orf42, C8orf37, CC2D2A, CCDC103, CCDC114, CCDC151, CCDC28B, CCDC39, CCDC40, CCDC65, CCNO, CDH23, CEP104, CEP120, CEP164, CEP290, CEP41, CEP83, CFAP300, CFAP410, CFTR, CLRN1, COL1A1, COL1A2, CRB1, CRB2, CRTAP, CRX, CSPP1, DCDC2, DDX59, DLL3, DNAAF1, DNAAF2, DNAAF3, DNAAF5, DNAH1, DNAH11, DNAH5, DNAH8, DNAH9, DNAI1, DNAI2, DNAJB13, DNAL1, DRC1, DYNC2H1, DYNC2LI1, DYX1C1, EVC, EVC2, FAM149B1, FKBP10, FOXH1, FOXJ1, FUZ, GALNT11, GAS2L2, GAS8, GLI3, GLIS2, GUCY2D, HES7, HYDIN, HYLS1, IFITM5, IFT122, IFT140, IFT172, IFT27, IFT43, IFT52, IFT57, IFT74, IFT80, IFT81, IMPDH1, INPP5E, INTU, INVS, IQCB1, KCNJ13, KIAA0556, KIAA0586, KIAA0753, KIF14, KIF7, KRAS, LBR, LCA5, LFNG, LRAT, LRRC56, LRRC6, LZTFL1, MAPKBP1, MCIDAS, MESP2, MKKS, MKS1, MYO7A, NEK1, NEK10, NEK8, NME8, NODAL, NPHP1, NPHP3, NPHP4, NRAS, OFD1, P3H1, PCDH15, PDE6D, PIBF1, PIH1D3, PKD1, PKD2, PKHD1, PPIB, PTPN11, RAF1, RD3, RDH12, RP1, RPE65, RPGR, RPGRIP1, RPGRIP1L, RSPH1, RSPH3, RSPH4A, RSPH9, SCLT1, SDCCAG8, SERPINF1, SERPINH1, SOS1, SP7, SPAG1, SPATA7, SUFU, TCTEX1D2, TCTN1, TCTN2, TCTN3, TEKT2, TMEM107, TMEM138, TMEM216, TMEM231, TMEM237, TMEM67, TOPORS, TRAF3IP1, TRIM32, TRIP11, TTC12, TTC21B, TTC8, TULP1, UMOD, USH1C, USH1G, USH2A, VHL, WDPCP, WDR19, WDR34, WDR35, WDR60, WHRN, XPNPEP3, ZIC3, ZMYND10, ZNF423*
(c) Genes involved in intestinal development or have been associated with IM that are not included in a and b (103 genes)
*ACTN1, ACVR2B, ADAM21, ALK, ATXN7, BAK1, BARX1, C16orf74, CCDC32, CDX1, CDX2, CDX4, CER1, COX16, CUTA, DAAM2, DAXX, DCAF17, DCAF5, DNAJC14, ERH, EXD2, FBXO31, FEZF2, FGF8, FZD4, FZD8, GALNT14, GALNT16, GGNBP1, GSE1, HESX1, HOXA4, HOXA5, HOXB5, HOXB6, HOXB7, HOXC12, HOXC13, HOXC4, HOXC5, HOXC6, HOXC8, HOXC9, HOXD10, HOXD11, HOXD12, HOXD13, HOXD4, HOXD8, HOXD9, HTR2A, IRF8, KAT6B, KCNMA1, LAMB2, LEFTY1, LEFTY2, LHX1, MAGI1, MAP2K2, MMP19, MNS1, MYCN, NKX2-5, NOTCH1, NOTCH4, NXPH4, OTX2, PDX1, PHF1, PITX2, PRICKLE1, PRICKLE2, PYM1, RAD51B, RBFOX1, SASH1, SFRP1, SFRP2, SHH, SLC6A4, SLC7A5, SMOC1, SOX2, SYNGAP1, SYNPR, TBX15, TBX18, TBX19, TBX2, TBX21, TBX22, TBX3, TBX4, TBX5, TBX6, TBXT, WDR37, WNT1, WNT5A, ZBTB9, ZFP36L1*
(d) Additional candidate genes identified through findings from Chromosomal Microarray Analysis in a previous study on patients with intestinal malrotation [19] (13 genes)
*CFL1, CFL2, DSTN, ELN, FBXO15, FOXC2, FOXL1, LIMK1, LIMK2, RHOA, ROCK1, ROCK2, RTTN*
(e) Genes that are interacting with or paralogues for TTC7A (22 genes)
*CAMKV, CFAP70, EFR3A, EFR3B, FAM126A, FAM126B, IFT88, OGT, PI4KA, PI4KB, PSMC1, RAB3IL1, RGS20, TMTC1, TMTC2, TMTC3, TMTC4, TTC13, TTC16, TTC34, TTC6, TTC7B*

## Results

### Participants

The cohort consisted of ten patients (nine males and one female) none of whom had any known malformations or comorbidities, except from one patient who was born at 35 + 0 weeks with intrauterine growth retardation. All had early symptoms, with seven diagnosed within the first month of life. Of the remaining three, two were diagnosed at one year of age and one was four years old at the time of diagnosis and subsequent surgery. All but one patent presented with midgut volvulus at diagnosis. The exception, who did not have volvulus at the initial surgery, needed redo-surgery due to recurrent midgut volvulus later.

### Sequencing data

**Fig. 1:**
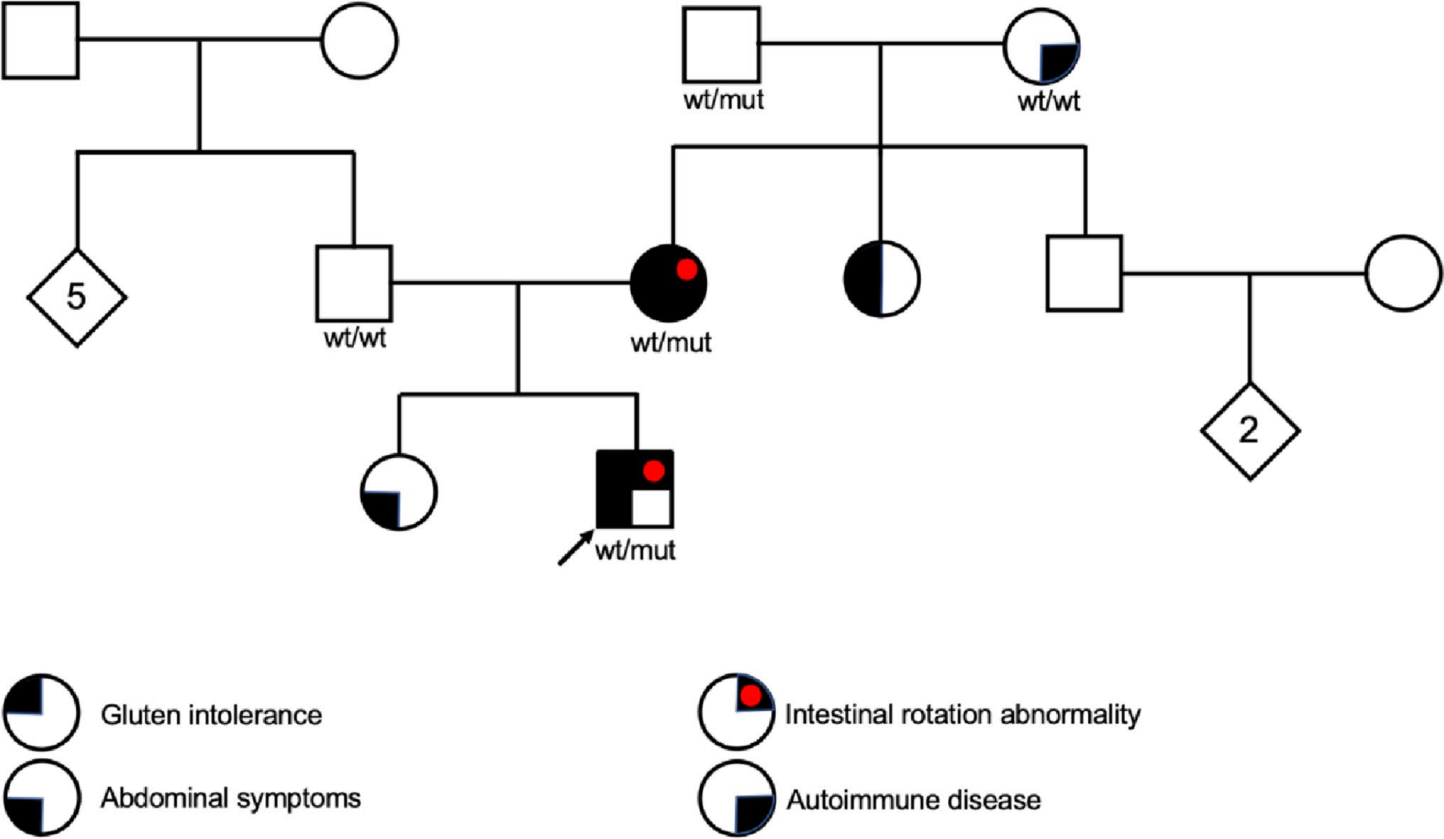
Pedigree of the proband’s family with two mutations in *cis* in the *TTC7A-*gene

In three patients, four genetic variants were identified (Table 3). The first patient had two heterozygous rare variants in *TTC7A* (NM_020458.4) c.433G > A p.(Ala145Thr) and c.1057G > A p.(Glu353Lys), located in exon 3 and exon 8 respectively. The variants were inherited from the mother and the maternal grandfather (Fig. 1). The second patient had a heterozygous missense variant in *ROCK2* (NM_004850): c.1802 A > T p.(Asp601Val). The third patient carried a missense variant in *LIMK2* (NM_016733): c.884 G > A p.(Arg295His). Seven patients had no variants of significance among the candidate genes. No SV:s were found.

**Table 3:** Identified variants of interest in this study when analyzing SNVs in a candidate gene panel (442 genes)

Patient	Gene	Variant		Inheritance	Allele frequency (GnomAD)	CADD-score	SIFT
P1	*TTC7A*	c.433G > A p.(Ala145Thr)	Missense, exonic	maternal	0.0002	22.8	Tolerated
P1	*TTC7A*	c.1057G > A p.(Glu353Lys)	Missense, exonic	maternal	0.000008	25.9	Damaging
P3	*ROCK2*	c.1802 A > T p.(Asp601Val)	Missense, exonic	paternal	0.00036	24.7	Damaging
P5	*LIMK2*	c.884 G > A p.(Arg295His)	Missense, exonic	paternal	0.00006	25.1	Damaging

### Clinical data of *TTC7A* variant carrier

**Fig. 2:**
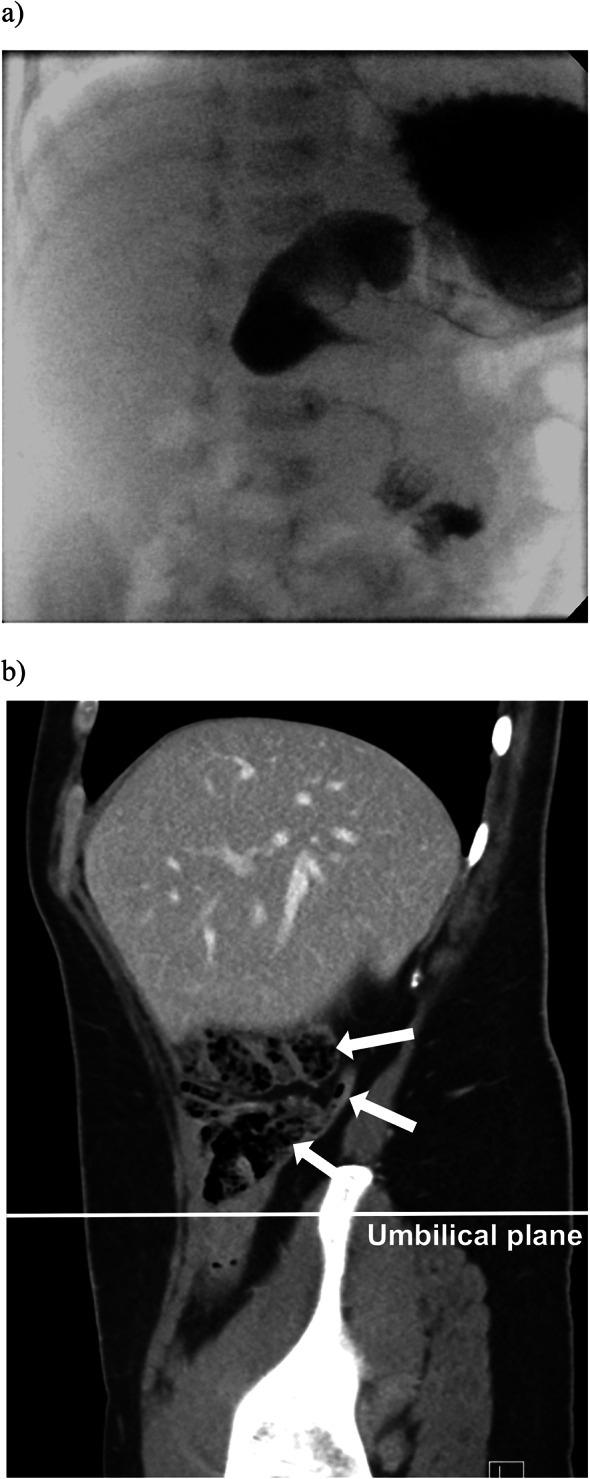
Radiological findings, patient 1 with two mutations in *cis* in the *TTC7A-*gene. **a** The proband. An upper gastrointestinal study, demonstrating IM with midgut volvulus, here with a “bird’s beak” and corkscrew appearance of the duodenum. **b** The mother. Computed tomography, a sagittal view demonstrating caecum in the right upper quadrant. Arrows, cranial to caudal direction: the ascending colon, appendix and caecum

The male proband underwent surgery for midgut volvulus when he was only 16 days old (Fig. 2a). Besides IM and clinically confirmed gluten intolerance, there were no other congenital malformations or comorbidities until the age of 14 years. Radiological investigations, previously performed on the mother, were reviewed by an experienced radiologist and showed a mild variant of IM, with the cecum fixated in the upper right quadrant, above the umbilicus (Fig. 2b). The superior mesenteric artery and vein were however positioned correctly indicating an atypical IM. The mother also had an autoimmune disorder. The maternal grandfather had no other gastrointestinal symptoms other than a bleeding gastric ulcer at the age of 40 years, and no immunological disorder. Ultrasound showed that the SMA was dislocated to the right, however an upper GI study could not confirm a diagnosis of IM.

### Clinical data of *ROCK2* variant carrier

The male patient had symptoms from birth and underwent surgery for midgut volvulus at four years of age. At 20, he is healthy apart from allergic asthma. The variant was inherited from the father who did not report any symptoms of intestinal malrotation and has not undergone clinical evaluation.

### Clinical data of *LIMK2* variant carrier

The boy underwent surgery for intestinal malrotation when he was one year old. After recurrent episodes of abdominal pain, vomiting as well as gastroesophageal reflux disease and faltering growth, he had re-do surgery for midgut volvulus at seven years. At age 23, he is healthy, with no other malformations or diseases. The variant was inherited from the father who had no symptoms of intestinal malrotation and has not undergone clinical evaluation.

All three patients had the duodenojejunal junction on the right side of the vertebral spine, Ladd’s bands, malpositioned caecum and a thin mesenteric base.

## Discussion

**Fig. 3:**
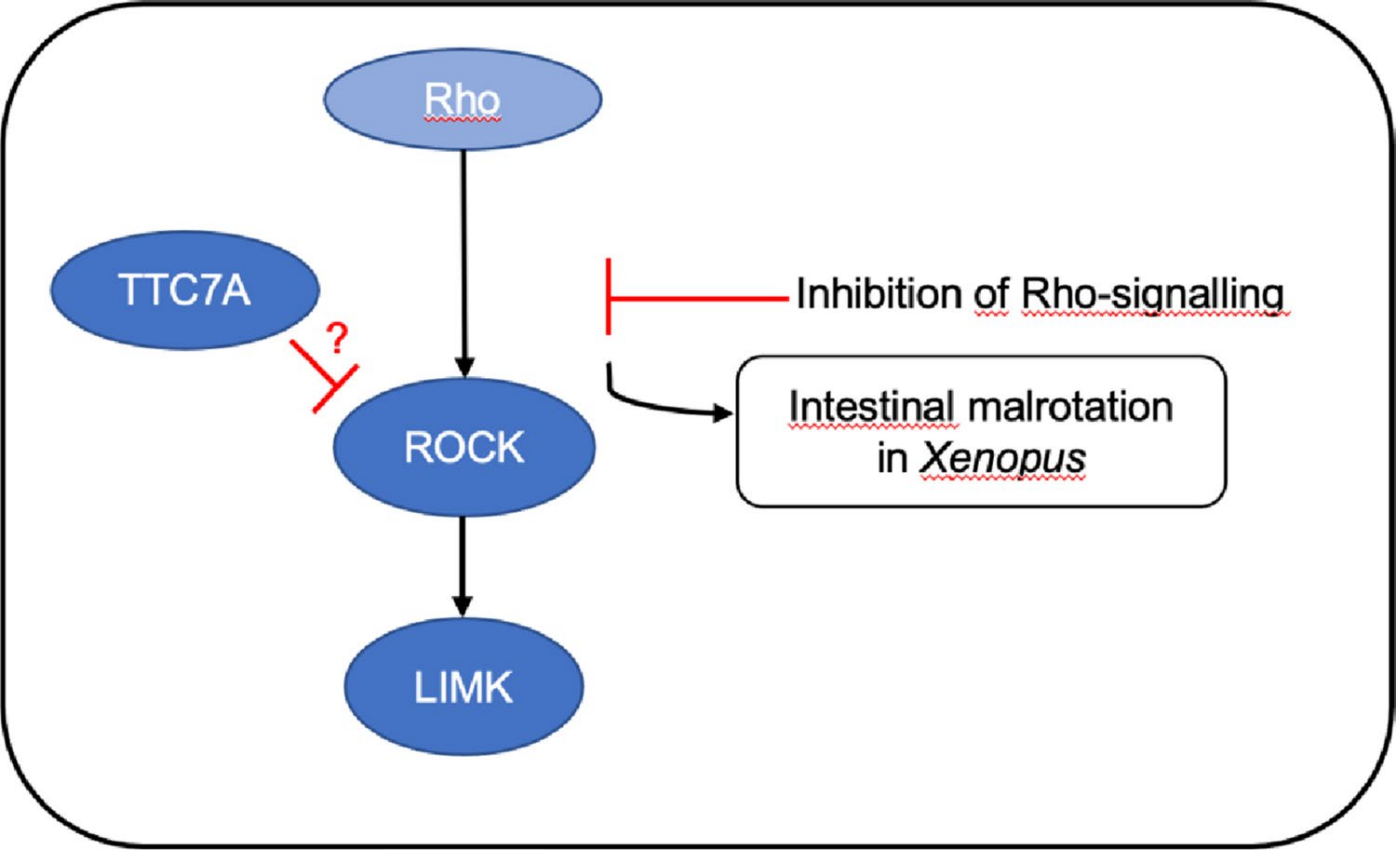
Rho/ROCK pathway involving *TTC7A*, *ROCK2* and *LIMK2*

In this pilot study of ten patients with isolated and early diagnosed IM, we identified one patient with two allelic variants in the *TTC7A-*gene, inherited from an affected mother. Two additional boys had variants in the *ROCK2* and *LIMK2* genes respectively. All three genes are part of a shared signaling pathway previously associated with intestinal malrotation, as illustrated in Fig. 3 and described below [27].

**Fig. 4:**
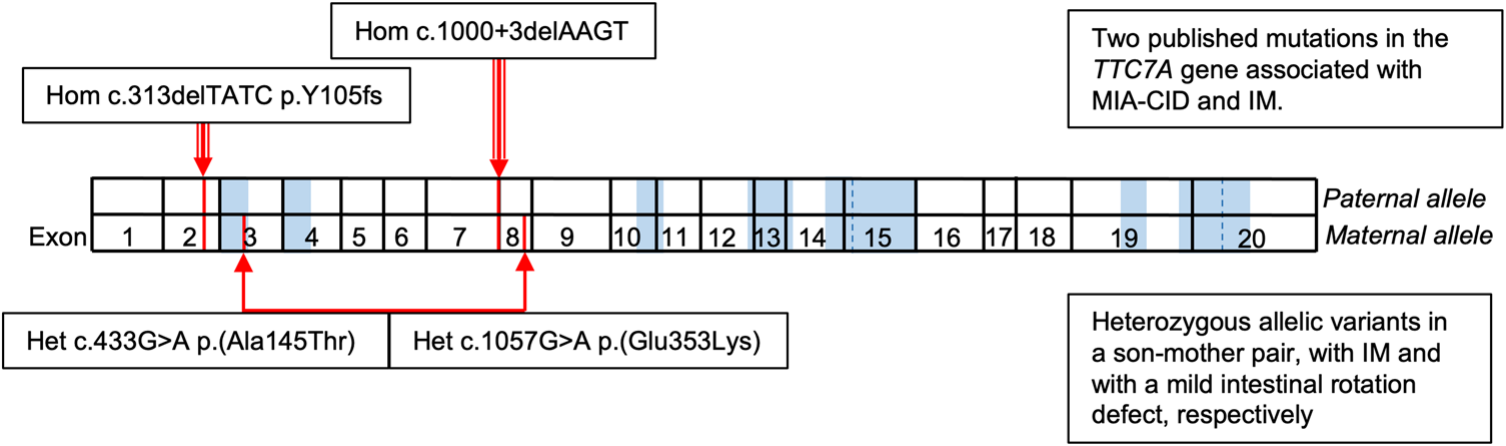
Variants in the *TTC7A*-gene in red previously reported in association with MIA-CID in combination with IM above the gene model [30, 31], and the variants reported in the present study below. TPR-domains are marked in blue

The *TTC7A-*gene*,* located on chromosome 2p21 with 20 exons plays a role in intestinal and spleen development. The protein contains nine tetratricopeptide repeat-domains (TPR-domains). Homozygous or compound heterozygous mutations lead to the autosomal recessively inherited Gastrointestinal defects and immunodeficiency syndrome (GDID, #243150) characterized by multiple intestinal atresias (MIA) in both the small and large intestines and has a poor prognosis. Some patients have immune defects or early onset inflammatory bowel disease. The severity of the GDID syndrome varies and generally missense mutations cause milder phenotypes while null mutations are associated with severe phenotypes [28]. Interestingly, to date, five patients with MIA have been reported to be associated with IM, initially three patients without genetic testing, one of which was secondary due to omphalocele [29]. Additionally, two case-reports highlighted MIA-CID (combined immunodeficiency) with IM and small homozygous deletions in *TTC7A*; c.53344_53347del [30] and c.313_216del [31]. Our report adds a third patient with *TTC7A* associated IM, however here both variants are in *cis* only affecting the maternally inherited allele (Fig. 4).

Further examination of the mothers’ phenotype revealed a mild intestinal rotation abnormality with a highly positioned caecum. This could easily have been overlooked without a detailed review of previous radiologic investigations, suggesting that familial cases could be more prevalent than previously understood. The mother also suffers from an autoimmune disorder affecting the muscles and skin, which is described as part of the clinical phenotype in TTC7A-deficiency.

*TTC7A* is a part of the Phosphatidylinositol 4-kinase IIIα-complex (PI4IIIα-complex), consisting of FAM126A/B-TTC7A-PI4IIIα-EFR3 proteins, and plays a critical role in the synthesis of phosphatidylinositol 4-phosphate (PI4P) within the cell membrane [32]. PI4P is important for maintaining cell membrane identity, polarity, cell survival and indirectly cell migration and proliferation [33]. Different alterations in PI4KIIIα (translating from *PI4KA*) may cause different diseases such as CNS alterations and MIA [34]. In one consanguineous Amish family, 13 patients carried a homozygous missense mutation in *PI4KA*, causing MIA, inflammatory changes in the intestines and IM in two patients [34]. Biochemical analysis demonstrated that the *PI4KA* c.4867 T > G p.(Tyr1623Asp) variant had a negative impact on the interaction between PI4KIIIα and TTC7A causing reduced complex formation, thus would primarily affect the tissues where the *TTC7A* gene is expressed. In the same study they also showed that *TTC7A* is expressed in a larger extent in the intestines than in the brain while *TTC7B* is mainly expressed in the brain [34]. These findings support the notion that the symptoms associated with TTC7A deficiency may be due to a defect complex formation and that IM may arise from a dysfunction in the PIKIIIα-complex.

In this study we identified two rare missense variants in *cis* in *TTC7A* located in exon 3 and 8 respectively. The exon 8 variant, c.1057G > A, is observed only twice in GnomAD and absent in the Swedish population (https://gnomad.broadinstitute.org/variant/2-47222330-G-A?dataset=gnomad_r2_1). The exon 3 variant, c.433G > A, is in a TPR-domain associated with a severe outcome [32]. This variant has been described previously and is annotated as of unknown significance in ClinVar (https://gnomad.broadinstitute.org/variant/2-47184062-G-A?dataset=gnomad_r2_1).

To date, 55 individuals with MIA have been reported with disease causing homozygous or compound heterozygous missense, nonsense and frameshift mutations in *TTC7A* [32]. Our analysis revealed two missense variants in *cis* suggesting an autosomal dominant form of *TTC7A* disease. A few examples in the literature supports this idea. One patient with MIA, IBD and immunodeficiency harbored a heterozygous *TTC7A* mutation inherited from the father (exon 20 c.2468 T > C p.Leu823Pro) and a maternally inherited mutation in the *CF1* gene (exon 4 c.500G > A, p.Arg167Lys), which in combination was interpreted to have caused the phenotype [35]. Furthermore, a study on 401 pediatric IBD-patients, five carried two heterozygote disease causing variants in the *TTC7A* gene in cis [36].

*ROCK2* located on chromosome 2p25.1 has 33 exons and is a key regulator in cell polarity, involved in smooth muscle contraction as well as actin cytoskeleton formation and focal adhesions. *ROCK2* is expressed in nearly all human tissues including the intestines and found in the ciliary proteome, important for the early stages of left–right (L–R) patterning during fetal development. CNVs involving ROCK2 have been associated with heterotaxy [37]. Additionally, knockdown of Rock2b via morpholino-modified antisense oligonucleotides in zebrafish affects the L-R patterning. The missense variant in this study is in a conserved region in most mammals but translates into Glutamate instead of Aspartic acid in zebrafish, chicken and xenopus. It is in the coiled coil region and has not previously been associated with disease.

*LIMK2* located on chromosome 22q12.2 has 18 exons and is expressed in many tissues including the intestines. It regulates actin cytoskeleton remodeling and contributes to cell migration. It is a part of the Rho-ROCK pathway and when activated, it phosphorylates cofilin, thus inhibiting its actin-depolarization activity. *LIMK2* also plays an important role in ciliogenesis [38]. The variant identified in this study is in a conserved region in all mammals as well as zebrafish, chicken and xenopus.

We have identified genetic variants in three genes that interact within the Rho-ROCK pathway (Fig. 3). LIMK2 acts as a key downstream effector of ROCK2 influencing actin polymerization by inhibiting cofilin. The TTC7A protein is also recognized but the specific function is not fully understood [39]. The Rho-ROCK pathway plays a vital role in the formation and elongation of the gut-tube in *Xenopus*. Studies using Rho kinase inhibitors during embryogenesis have shown that mild interference leads to IM, while more significant disruptions result in elongation deformities [27]. Moreover, other studies indicate that mechanical forces drive actin assembly through the Rho-ROCK-LIMK-cofilin pathway [40] and that a mechanically driven growth mechanism is active during intestinal development and rotation [41]. Notably, a loss of function mutation in filamin-A (*FLNA*) causes a syndrome with short bowel and IM [21, 22] and FLNA regulates Rho [42], linking back to the Rho-ROCK pathway.

**Fig. 5:**
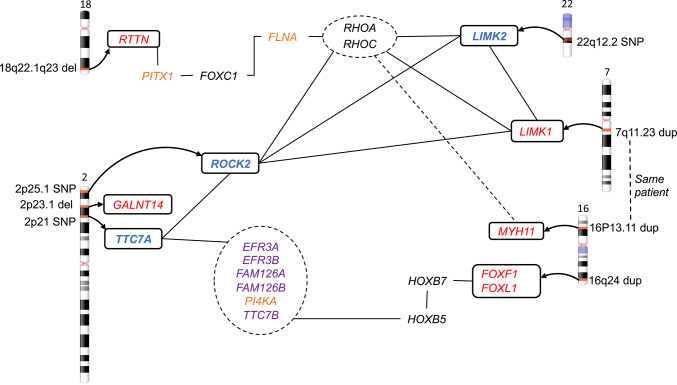
Gene map with network data from String on genes recognized in this study (marked in blue) as well as interesting genes located within CNVs in patients with IM (marked in red) [19]. Genes marked in orange are strongly associated with IM and genes marked in purple belong to a protein complex

In Fig. 5 we summarize the findings of genes from this study, and from our previous study on CNVs in IM [19] and show associations as described [26] to connect genes related to IM. *PITX1*, one of the important genes in left–right patterning and *FLNA*, earlier mentioned, link an interesting gene, *RTTN*, located in the region of the 18q-deletion syndrome, to the Rho/ROCK-pathway. *LIMK1* is in a 7q11.23 duplication in two patients with IM [19, 43] and interacts with the Rho/ROCK-pathway. The protein complex that *TTC7A* belongs to, is related to HOXB-genes, interacting with *FOXF1*. Mutations in *FOXF1* cause alveolar capillary dysplasia with misalignment of pulmonary veins and IM [44]. Duplications involving FOXF1 cause intellectual disability, speech delay and gastrointestinal malformations including IM [19, 44]. The patient with a 7q11.23 duplication also carried a 16p13.11 duplication with unknown significance, containing the *MYH11* gene. Mutations in this gene cause Megacystis-microcolon-intestinal hypoperistalsis syndrome 2, an autosomal recessive disorder. MYH11 is active in regulation of cytoskeleton remodeling by Rho GTPases.

A key strength of this study is access to detailed phenotypic data, extra important in IM due to the variable presentation. IM is a diagnosis that can easily be overlooked, and familial IM can be difficult to identify due to non-specific or absent symptoms. Isolated IM might be caused by environmental factors or complex genetic factors and is most likely not a monogenic condition in most cases. A limitation is the small number of patients investigated, partially explained by a relatively rare condition with strict inclusion criteria. In the future, studies are needed to investigate the Rho-ROCK pathway in a larger cohort of isolated IM cases.

## Conclusions and future directions

By Genome sequencing of patients with early-onset isolated IM, we identify potential genetic contributors. We show that heterozygous variants in *TTC7A*-gene may cause dominant IM. Furthermore, the detection of missense variants in the *ROCK2* and *LIMK2* genes in two other patients highlights the significance of the Rho-ROCK signaling pathway in intestinal development and its potential disruption in IM. This is a pilot study, and the findings can be incidental and need to be analyzed in a larger setting by screening for sequence variants, optimally GS in trios with isolated IM to identify de novo mutations and autosomal recessive disorders associated with IM.

## Data Availability

All data supporting the findings in this study are available within the paper or upon request from the authors.
